# Time trends and geographical variation in major lower limb amputation related to peripheral arterial disease in England

**DOI:** 10.1093/bjsopen/zrad140

**Published:** 2024-01-05

**Authors:** Ravi Maheswaran, Thaison Tong, Jonathan Michaels, Paul Brindley, Stephen Walters, Shah Nawaz

**Affiliations:** School of Health and Related Research, University of Sheffield, Sheffield, UK; School of Health and Related Research, University of Sheffield, Sheffield, UK; School of Health and Related Research, University of Sheffield, Sheffield, UK; Department of Landscape Architecture, University of Sheffield, Sheffield, UK; School of Health and Related Research, University of Sheffield, Sheffield, UK; Sheffield Vascular Institute, Sheffield Teaching Hospitals NHS Foundation Trust, Sheffield, UK

## Abstract

**Background:**

Above and below knee amputation (AKA, BKA) are treatments of last resort for peripheral arterial disease (PAD). The aim was to examine amputation rates, AKA:BKA ratios, previous revascularization and minor amputation, lengths of stay in hospital, mortality following amputation, and regional variation in people with and without diabetes in England.

**Methods:**

The study used population-based ecological and cohort study designs, 31 672 census areas, hospital admissions from 2006 to 2018 and Poisson, logistic and Cox regression.

**Results:**

There were 47 249 major lower limb amputations (50.7% AKA; 48% had diabetes), giving an annual PAD-related amputation rate of 11 per 100 000 in the population aged 25+ years. Amputation rates were higher in men and substantially higher in people with diabetes. The AKA:BKA ratio was 0.63 in patients with diabetes (*n* = 22 702) and 1.62 in patients without diabetes (*n* = 24 547). Of patients having AKA, 25.3% died within 90 days of amputation compared with 11.9% for BKA. Median survival following amputation ranged from only 1.68 years following AKA in patients with diabetes to 5.72 years following BKA in patients without diabetes. Amputation rates decreased over time mainly in the population with diabetes. Short-term mortality and lengths of stay in hospital also decreased over time, while the percentage with previous revascularization generally increased. Amputation rates and AKA:BKA ratios were highest in the North. Adjustment for age, sex and deprivation did not substantially alter geographical patterns. Adjusted 90-day mortality was generally higher in the North and the Midlands but also high in London. There were also regional variations in adjusted duration from admission to amputation, duration from amputation to discharge or death in hospital, previous revascularization and previous minor amputation.

**Conclusions:**

There were large variations in amputation rates and survival following amputation in relation to diabetes status and amputation level, and regional variations which remained after adjustment for deprivation.

## Teaser

The study used population-based ecological and cohort study designs, 31 672 census areas, hospital admissions from 2006 to 2018 and Poisson, logistic and Cox regression to examine time trends and geographical variation in PAD-related major amputation in England. There were clear contrasting patterns in relation to diabetes status, and marked geographical variations in quality indicators for major amputation. Further action is required to reduce amputation rates and improve survival following amputation.

## Introduction

Peripheral arterial disease (PAD) can be a debilitating condition and major amputation of the lower limb is reserved as a treatment of last resort when other options do not exist or have failed^1^. Internationally, reported amputation rates appear to vary widely^2,3^. In addition, major amputation rates are much higher in people with diabetes^4–6^. Geographical variation in major amputation rates also exists within countries, including the USA and England^1,5,7^, and differences in healthcare delivery could contribute to this variation.

Mortality following major amputation is high^1,8,9^, and guidance has been proposed to improve outcomes. For example, the Vascular Society for Great Britain and Ireland produced guidelines for a best practice pathway while the UK All-Party Parliamentary Group on Vascular and Venous Disease issued a call for action to reduce inequalities in major amputation rates^10,11^.

While a number of studies have reported on major lower limb amputation rates in England^4–8,12–14^, including some that have examined trends over time^4,6,8,12^, few have examined trends over time in mortality following amputation^8^. Major lower limb amputation is typically performed as above knee amputation (AKA) or below knee amputation (BKA). The National Vascular Registry annual reports provide AKA and BKA counts and AKA:BKA ratios and include data from hospitals in England^15^. However, few published studies have examined AKA and BKA rates or ratios over time in England^8,12^.

The aims of this national population-based study of PAD-related major lower limb amputation in England were to investigate (i) trends over time in AKA and BKA rates in people with and without diabetes; (ii) trends over time in previous procedures (revascularization; minor amputation), lengths of stay in hospital before and after amputation, and mortality following amputation and (iii) geographical variation in amputation rates and in these quality indicators.

## Methods

### Hospital episode statistics data on hospital admissions and mortality

Hospital episode statistics (HES) in-patient data on admissions to NHS hospitals in England from April 2006 to March 2018, with linked mortality records, were provided by NHS England, which manages all NHS hospital data in England. All patients who had AKA or BKA were identified using Office for Population Censuses and Surveys (OPCS) procedure codes, the standard classification system used in the English National Health Service (NHS)^16^. All traumatic and malignant amputations identified using trauma-related and malignancy-related ICD-10 codes^17^ in the main diagnosis field were excluded.

Pseudo-anonymized patient identifiers were used to link all admissions for each patient. The index admission was defined as the first admission with AKA or BKA and the data set was restricted to patients aged 25 years or older.

Patients with diabetes were identified using ICD-10 codes in the first 14 diagnosis fields in the index admission or in any previous admission within one year of the index admission. Co-morbidities were also identified using this approach. Seven co-morbidity categories were considered (see results), based on the Royal College of Surgeons Charlson Score and opinions from the expert clinical advisory panel for this project^18,19^.

The pre-amputation pathway was examined in terms of previous procedures (endovascular or open revascularization for PAD; minor amputation) within one year preceding the index admission. Lengths of stay in hospital were examined in terms of the delay between the date of admission and the operation date and the length of stay in hospital following the operation. Short-term mortality was examined as in-hospital deaths, and deaths within 90 days, following amputation.

### Study design, geography and population data

A population-based (ecological) study design was used to examine amputation rates and a cohort study design was used to examine mortality following amputation.

Lower layer super output areas (LSOAs) were used as the basic geographical units. These are census areas with an average population of 1500 people^20^. As there were a small number of changes to LSOAs between the 2001 Census and the 2011 Census, the analysis was restricted to 31 672 of the 32 844 LSOAs in 2011 (96.4%) to maintain consistency across the study time span.

LSOA mid-year population estimates by age and sex were used and aggregated as appropriate to calculate amputation rates by age and sex. Diabetes prevalence estimates by age and sex from the Health Survey for England^21^ were used to calculate estimates of the national population with and without diabetes.

Geographical analysis used the nine Standard Regions in England^22^. The Income Domain from the Index of Multiple Deprivation 2010 was used to adjust for differences in socioeconomic deprivation in regional comparisons^23^.

### Statistical analysis

Population-based rates by age and sex and trends over time are presented as graphs. Population-based rates were compared using Poisson regression. Logistic regression was used to compare percentages of patients with co-morbidities, previous interventions and short-term mortality (in-hospital death, and death within 90 days, following amputation). Lengths of stay were examined using generalized linear modelling with a log-link transformation. Long-term survival was examined using Kaplan–Meier curves and Cox proportional hazards modelling, with follow-up to 31 March 2018. Results are presented as adjusted rate ratios, Odds ratios and hazard ratios with 95% c.i.

## Results

There were a total of 47 249 PAD-related major lower limb amputations in the 12-year period examined (50.7% AKA) in a population aged 25+ years of 35.7 million, giving an overall annual PAD-related major lower limb amputation rate of 11 per 100 000 in people aged 25 years or more.

It was estimated that an average of 9.5% of the 25+ population had diabetes during the time period examined. Of all major PAD-related amputations performed, 48% were on patients with diabetes. In patients with diabetes, 61.4% of amputations were carried out as BKA while in patients without diabetes 38.2% were carried out as BKA.

The overall AKA:BKA ratio was 1.03. This ratio was 0.63 in patients with diabetes and 1.62 in patients without diabetes.

The overall annual PAD-related major lower limb amputation rate in the 25+ population was 55.8 per 100 000 population in people with diabetes and 6.3 per 100 000 population in people without diabetes, giving an overall unadjusted rate ratio of 8.86. This rate ratio, adjusted for age, sex and year, for major PAD-related lower limb amputation in people with diabetes, relative to people without diabetes, was 4.82 (95% c.i. 4.73–4.91).

### Characteristics of patients

*Table 1* shows the characteristics of patients who had PAD-related major lower limb amputation (AKA and BKA).

**Table 1:** Characteristics and short-term mortality for the (*N* = 47 249) patients aged 25+ years who had PAD-related major lower limb amputation (AKA and BKA); England (April 2006 to March 2018)

Characteristic	Patients with diabetes		Patients without diabetes		Adjusted ratios (95% c.i.)	
	AKA (*n* = 8759)	BKA (*n* = 13 943)	AKA (*n* = 15 176)	BKA (*n* = 9371)	With *versus* without diabetes	AKA *versus* BKA
Age (years), mean(s.d.)	71.8(11)	66.1(12.4)	72.1(13.3)	65.1(15.8)		
Men (%)	64	76	61	69		
Average annual population	3 387 354	3 387 354	32 344 426	32 344 426		
Average annual rate (per 100 000)	21.55	34.30	3.91	2.41		
**Co-morbidities (%)**						
Coronary artery disease	47.2	40.6	31.0	23.8	2.08 (2.00,2.17)	1.02 (0.98,1.06)
Heart failure	27.6	22.3	18.0	11.5	1.94 (1.85,2.04)	1.06 (1.01,1.11)
Cerebrovascular disease	16.9	11.0	12.9	7.5	1.43 (1.35,1.51)	1.42 (1.34,1.51)
COPD	24.1	17.7	25.8	21.4	0.83 (0.8,0.87)	1.39 (1.33,1.46)
Renal disease	31.3	33.0	14.6	12.0	3.08 (2.94,3.23)	0.71 (0.68,0.74)
Cancer	6.5	4.6	8.0	6.5	0.73 (0.67,0.79)	1.29 (1.20,1.40)
Moderate or severe liver disease	1.1	0.9	1.1	1.1	0.96 (0.80,1.16)	1.50 (1.25,1.80)
**Previous intervention (%)**						
Previous revascularization	40.8	41.0	37.0	39.4	1.07 (1.02,1.11)	0.84 (0.80,0.87)
Previous minor amputation	13.4	35.1	5.0	12.7	3.20 (3.02,3.4)	0.32 (0.30,0.34)
**Length of hospital stay (days), median**						
From admission to amputation	8	8	6	5	1.19 (1.13,1.25)	1.04 (0.99,1.09)
From amputation to discharge or death	28	26	24	21	1.11 (1.09,1.14)	1.03 (1.01,1.05)
**Short term mortality (%)**						
In-hospital death	20.2	10.0	19.8	9.3	0.88 (0.83,0.93)	1.90 (1.79,2.01)
90-day mortality	25.6	12.4	25.2	11.2	0.91 (0.87,0.96)	2.03 (1.92,2.14)

AKA, above knee amputation; BKA, below knee amputation; COPD, chronic obstructive pulmonary disease.

A higher proportion of patients having amputation were men. This was more pronounced for BKA than for AKA, especially in patients with diabetes where 76% of patients having BKA were men.

The mean ages of patients with and without diabetes who had AKA were similar (71.8 (s.d. 11.0) years and 72.1 (s.d. 13.3) years, respectively). Likewise, the mean ages of patients with and without diabetes who had BKA were similar (66.1 (s.d. 12.4) years and 65.1 (s.d. 15.8) years, respectively). These figures also show that patients who had AKA were older than patients who had BKA.

With regard to co-morbidities, patients with diabetes had a higher prevalence Odds ratio (adjusted for age, sex and year) for coronary artery disease, heart failure, cerebrovascular disease and renal disease and a lower prevalence Odds ratio for chronic obstructive pulmonary disease (COPD) and cancer, relative to patients without diabetes.

Patients with AKA had higher prevalence Odds ratios for cerebrovascular disease, COPD, cancer and liver disease and a lower prevalence Odds ratio for renal disease relative to patients with BKA.

Overall, 39.4% of patients had had previous revascularization for PAD before amputation. The adjusted Odds of having had previous revascularization was marginally higher in patients with diabetes than in patients without diabetes (Odds ratio 1.07 (95% c.i. 1.02 to 1.11)) and lower in patients with AKA than in patients with BKA (Odds ratio 0.84 (95% c.i. 0.80 to 0.87)).

Of the patients who had BKA, 35.1% of patients with diabetes had previously had a minor amputation whereas only 12.7% of patients without diabetes had previously had a minor amputation. Comparatively fewer patients with AKA had previously had a minor amputation (13.4% in patients with, and 5.0% in patients without, diabetes).

Median duration from admission to amputation ranged from 5 to 8 days and was marginally longer for patients with diabetes. Median duration of stay following amputation was 28 and 26 days for AKA and BKA, respectively, in patients with diabetes and shorter at 24 and 21 days for AKA and BKA, respectively, for patients without diabetes.

### Amputation rates by age and sex

**Fig. 1:**
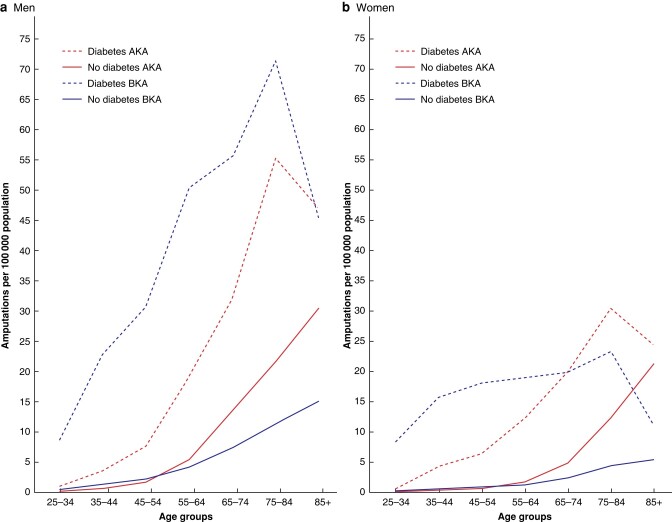
**Population-based AKA and BKA rates by age, sex and diabetes status; England (April 2006 to March 2018)** AKA, above knee amputation; BKA, below knee amputation.

*Figure 1* shows population-based PAD-related major lower limb amputation rates by age and sex.

AKA and BKA rates generally increased with increasing age. Amputation rates were generally higher in men than women, and clearly higher in people with diabetes than in people without diabetes.

In the population with diabetes, men had higher BKA than AKA rates except in the 85+ age group, where rates were similar. However, in women with diabetes, while BKA rates were higher than AKA rates in those aged 25–64 years, and similar in those aged 65–74 years, AKA rates were noticeably higher than BKA rates in women with diabetes aged 75–84 and 85+ years.

In the population without diabetes, AKA rates were clearly higher from the age of 65 years onwards in both men and women, with a greater increase with increasing age, compared with BKA rates.

### Short-term mortality following amputation

The unadjusted percentages of deaths (*Table 1*) were broadly similar in patients with and without diabetes (20.2% and 19.8%, respectively, for AKA and 10.0% and 9.3% for BKA). However, adjustment for age, sex, year, level of amputation and co-morbidities showed that short-term mortality was lower in patients with diabetes than in patients without diabetes (Odds ratio of 0.88 (95% c.i. 0.83 to 0.93) for in-hospital death and 0.91 (95% c.i. 0.87 to 0.96) for 90-day mortality). This was mainly as a result of adjustment for the prevalence of heart failure and of renal disease.

The main differences in short-term mortality (both in-hospital death and 90-day mortality following operation) were between AKA and BKA. Mortality was substantially higher in patients who had AKA, with 25.3% dying within 90 days of the operation, compared with 11.9% for BKA.

The Odds ratios for short-term mortality for AKA relative to BKA, adjusted for age, sex, year and co-morbidities, was 1.90 (95% c.i. 1.79 to 2.01) for in-hospital death and 2.03 (95% c.i. 1.92 to 2.14) for 90-day mortality.

### Long-term survival following amputation

**Fig. 2:**
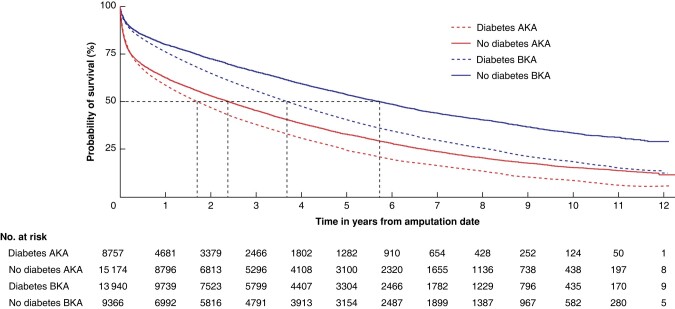
**Survival following major amputation in patients aged 25+ years by level of amputation (AKA or BKA) and diabetes status; England (April 2006 to March 2018)** AKA, above knee amputation; BKA, below knee amputation.

*Figure 2* shows overall survival curves following AKA and BKA for all patients aged 25+ years by diabetes status. Patients who had AKA had worse survival than patients who had BKA. In patients who had AKA, those with diabetes had worse survival than those without diabetes. Similarly, in patients who had BKA, those with diabetes had worse survival than those without diabetes.

*Table 2* shows the corresponding overall median survival times for all patients aged 25+ years. The shortest median survival time was for patients with diabetes who had AKA, which was only 1.68 years. Median survival for patients without diabetes who had AKA was 2.38 years. Median survival following BKA was 3.68 years in patients with diabetes and 5.72 years in patients without diabetes.

**Table 2:** Survival time and mortality hazard ratios (adjusted for age, sex and year) following PAD-related AKA and BKA in patients aged 25+ years with, and without, diabetes; England (April 2006 to March 2018)

Patient categories	Patients	Deaths	Median survival (years)
AKA in patients with diabetes	8757	6500	1.68
BKA in patients with diabetes	13 940	8400	3.68
AKA in patients without diabetes	15 714	10 403	2.38
BKA in patients without diabetes	9366	4648	5.72
**Comparisons between categories**			**HR (95% c.i.)**
AKA *versus* BKA (with additional adjustment for diabetes status)			1.39 (1.36,1.42)
Diabetes *versus* no diabetes (with additional adjustment for amputation level)			1.20 (1.17,1.23)
AKA *versus* BKA in patients with diabetes			1.36 (1.32,1.41)
AKA *versus* BKA in patients without diabetes			1.44 (1.39,1.49)

PAD, peripheral arterial disease; AKA, above knee amputation; BKA, below knee amputation.

*Table 2* also shows the hazard ratios for mortality following amputation, adjusted for age, sex and year. Patients with diabetes had 20% (95% c.i. 17 to 23%) higher mortality than patients without diabetes (hazard ratio also adjusted for level of amputation). Patients having AKA had 39% (95% c.i. 36 to 42%) higher mortality than patients having BKA (hazard ratio also adjusted for diabetes status). This percentage increase in the hazard ratio was broadly similar in patients with and without diabetes.

### Trends over time

**Fig. 3:**
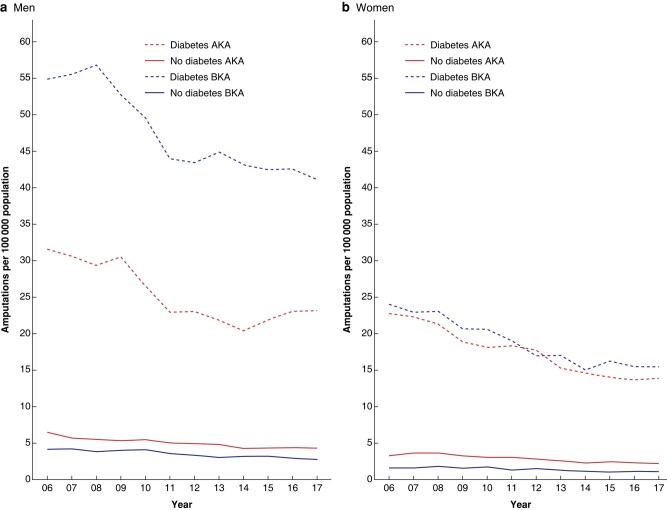
**Time trends in AKA and BKA rates by sex and diabetes status in the population aged 25+ years; England (April 2006 to March 2018)** AKA, above knee amputation; BKA, below knee amputation.

*Figure 3* shows trends over time from 2006/07 to 2017/18 in population-based PAD-related major lower limb amputation rates by sex for all people aged 25+ years combined. In the population with diabetes, both AKA and BKA rates generally decreased over time. In the population without diabetes, rates were much lower with small decreases over time.

**Fig. 4:**
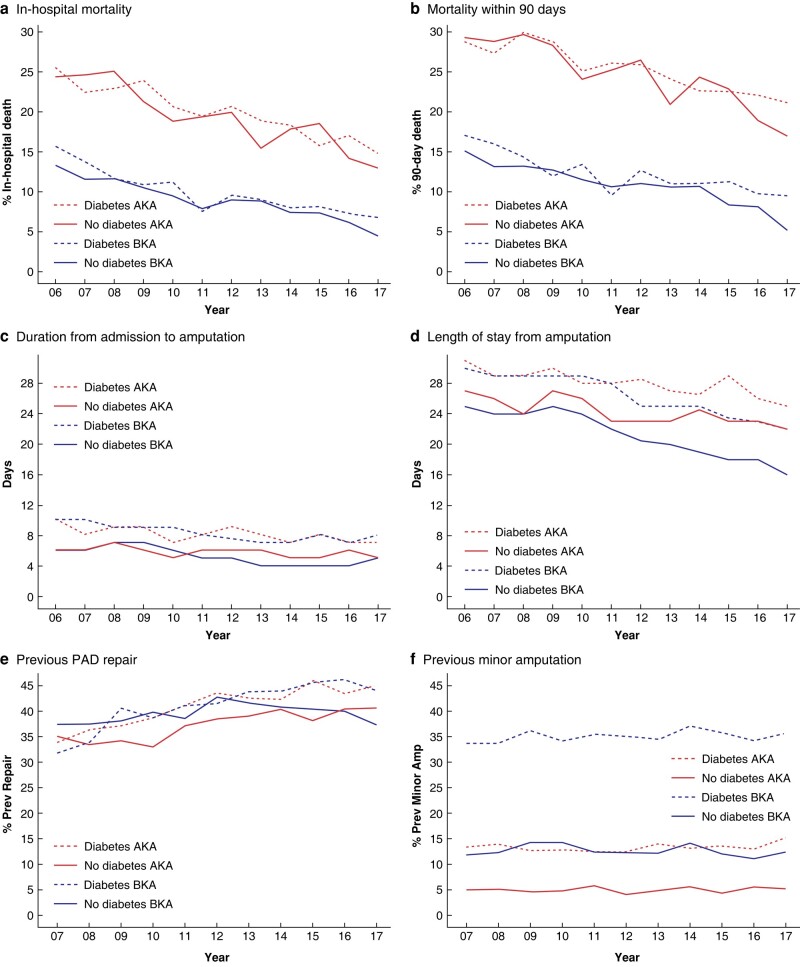
**Time trends in short-term mortality, duration of stay in hospital and previous procedures in patients aged 25+ years who had major lower limb amputation; England (April 2006 to March 2018)** PAD, peripheral arterial disease; AKA, above knee amputation; BKA, below knee amputation.

*Figure 4* shows trends over time in short-term mortality, duration of hospital stay and previous interventions. Short-term mortality decreased over time, duration from admission to amputation decreased slightly over time, and length of stay from amputation to discharge or death in hospital also decreased over time. There were no marked differences in trends in patients with and without diabetes. The decrease in length of stay after amputation was, however, greater for BKA than for AKA.

The percentage with previous revascularization for PAD increased over time in patients with AKA, but there was no marked difference in trends between patients with and without diabetes. For BKA, although there was an increase in this percentage in patients without diabetes, it reached a plateau from around 2012/13 onwards. In patients with diabetes, however, the pattern of increase appeared to continue.

The percentages with previous minor amputation tended to remain relatively unchanged over the time period examined. This was the case for both BKA and AKA in patients with and without diabetes.

### Regional variation

**Fig. 5:**
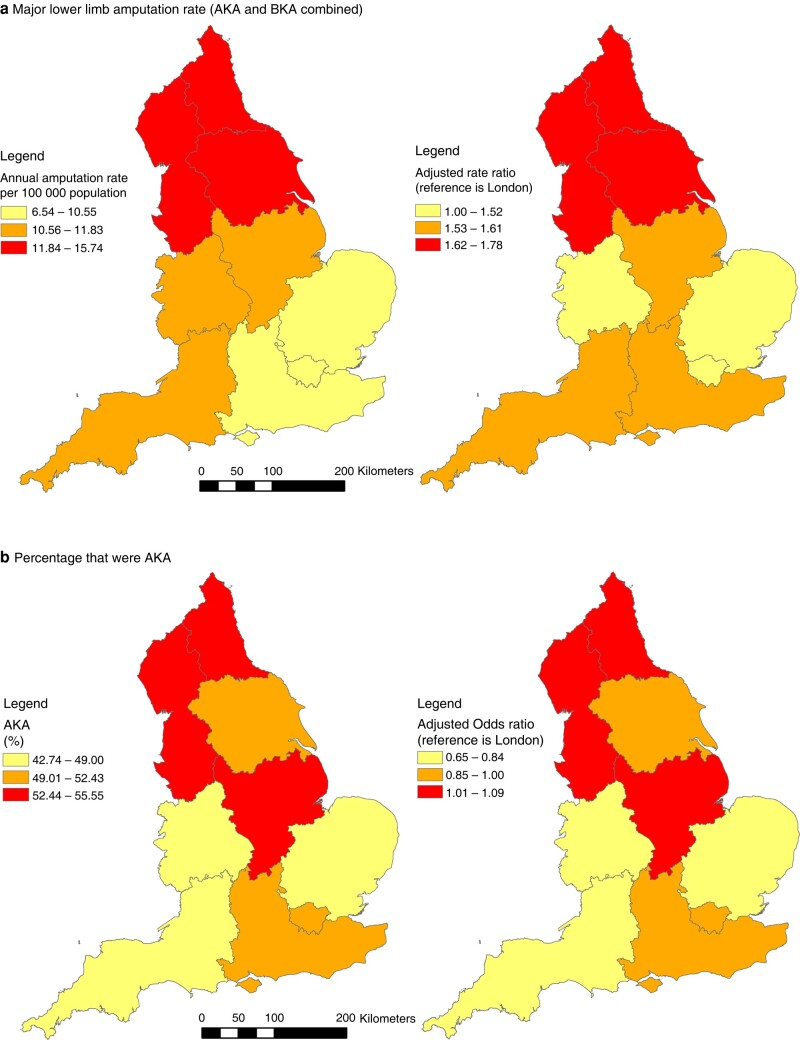

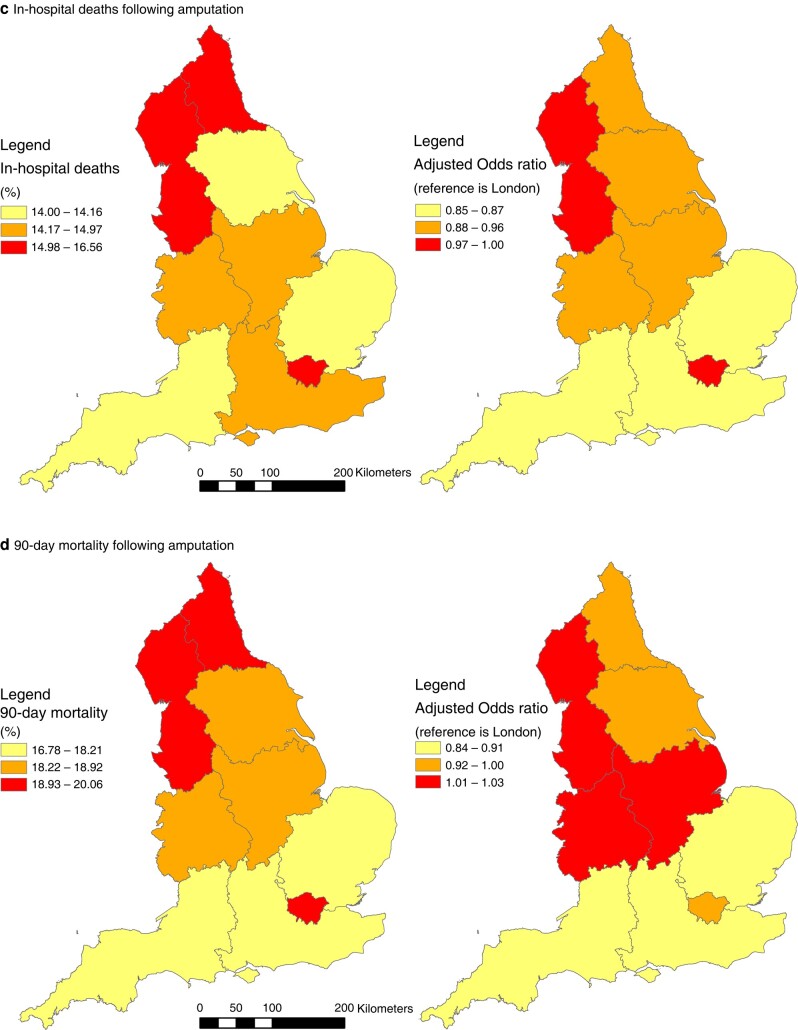

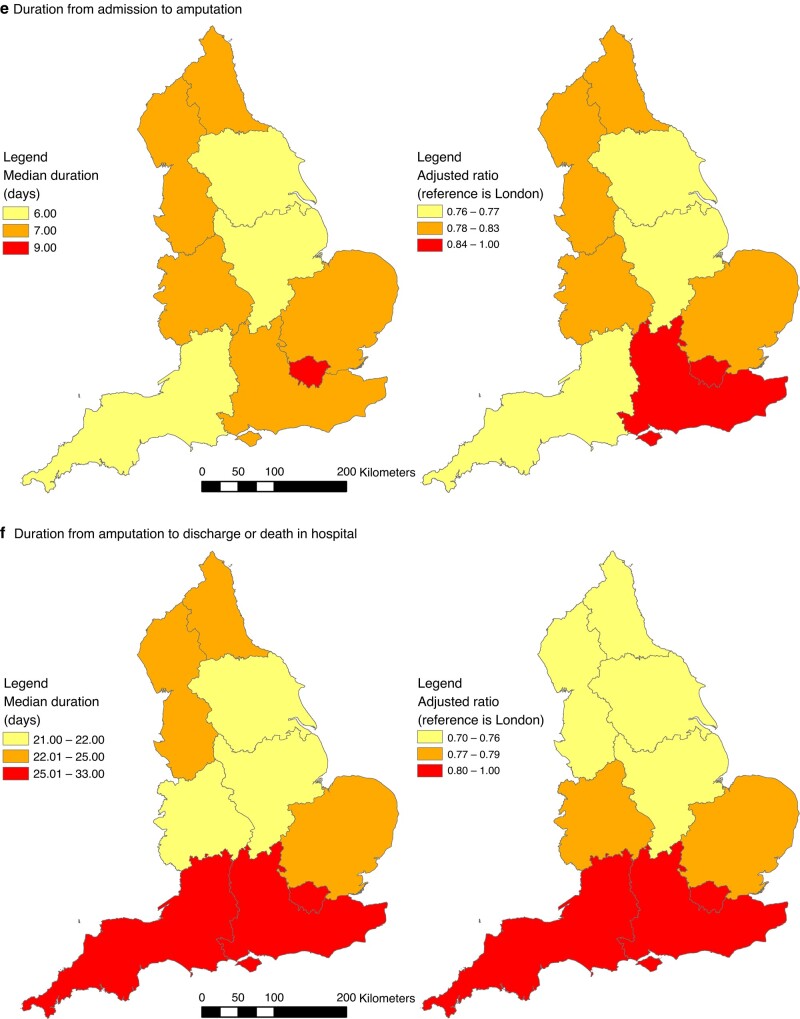

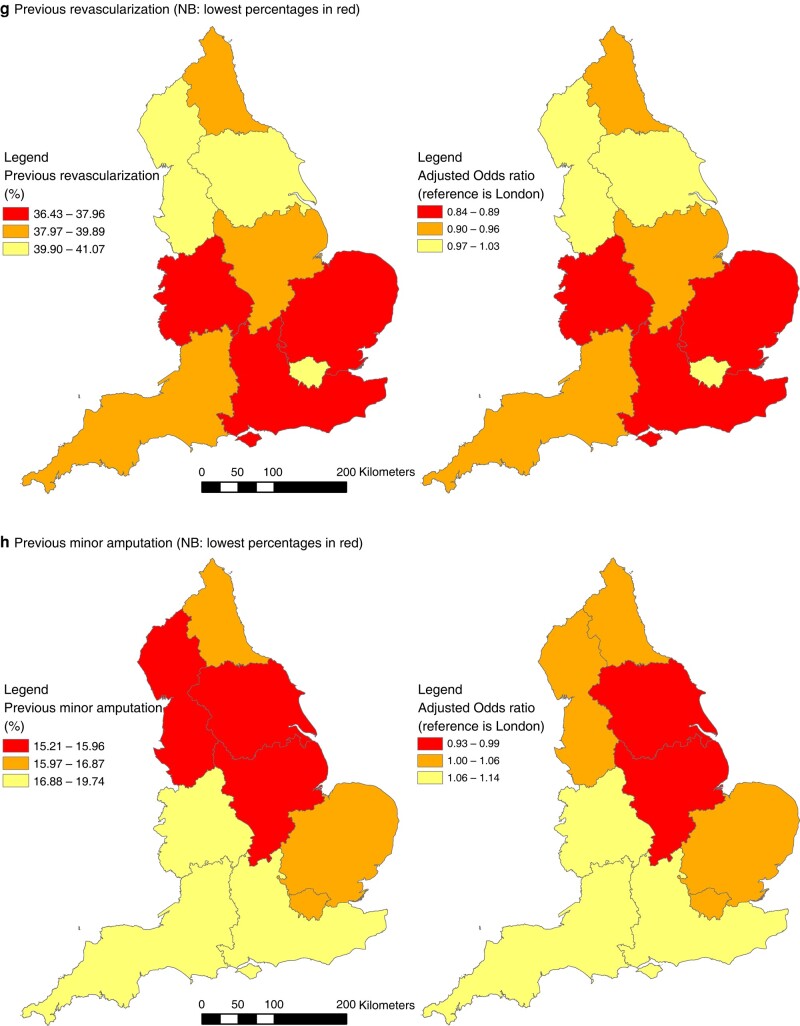
a–h Regional variation in major lower limb amputation rates, short-term mortality following amputation, duration of stay in hospital and previous procedures preceding amputation; England (April 2006 to March 2018) ‘Left panel’ shows unadjusted values; ‘Right panel’ shows adjusted ratios with London as the reference region. The ‘worst’ values are in red for all quality indicators. AKA, above knee amputation; BKA, below knee amputation.

Geographical variation in major amputation rates for the population aged 25+ years, and in quality indicators associated with amputation, is shown in *Table 3* and *Fig. 5* using Standard Regions in England. Adjusted ratios, with London as the baseline category, are also shown. The ‘worst’ regions are in red in *Fig. 5* for all quality indicators.

**Table 3:** Regional variation in major lower limb amputation rates, short-term mortality following amputation, duration of stay in hospital and previous procedures preceding amputation; England (April 2006 to March 2018)

Region	Major amputations		Percentage that were AKA	
	Annual rate per 100 000	Adjusted rate ratio (95% c.i.)*	Per cent	Adjusted Odds ratio (95% c.i.)*
London	6.5	1	52.4	1
East Midlands	11.8	1.61 (1.54,1.68)	52.7	1.03 (0.94,1.13)
East of England	10.5	1.52 (1.46,1.58)	49.0	0.84 (0.77,0.92)
North East	15.7	1.78 (1.70,1.86)	55.5	1.09 (0.99,1.20)
North West	14.0	1.74 (1.67,1.80)	55.5	1.08 (1.00,1.17)
South East	9.9	1.53 (1.47,1.59)	50.3	0.90 (0.83,0.98)
South West	11.8	1.59 (1.53,1.66)	42.7	0.65 (0.59,0.70)
West Midlands	10.9	1.36 (1.30,1.41)	47.4	0.83 (0.76,0.91)
Yorkshire and The Humber	12.6	1.62 (1.56,1.69)	50.1	0.92 (0.84,1.00)

Region	In-hospital deaths		90-day mortality	
	Per cent	Adjusted Odds ratio (95% c.i.)*	Per cent	Adjusted Odds ratio (95% c.i.)*
London	16.6	1	20.0	1
East Midlands	15.0	0.96 (0.85,1.08)	18.9	1.01 (0.90,1.13)
East of England	14.0	0.85 (0.76,0.96)	17.9	0.90 (0.80,1.00)
North East	15.9	0.96 (0.85,1.10)	19.5	0.98 (0.87,1.11)
North West	15.9	0.97 (0.87,1.08)	20.1	1.03 (0.93,1.14)
South East	14.4	0.87 (0.78,0.97)	18.2	0.91 (0.82,1.01)
South West	14.2	0.87 (0.77,0.98)	16.8	0.84 (0.75,0.94)
West Midlands	14.3	0.95 (0.84,1.07)	18.5	1.02 (0.92,1.15)
Yorkshire and The Humber	14.1	0.90 (0.80,1.01)	18.5	1.00 (0.90,1.12)

Region	Duration from admission to amputation		Duration from amputation to discharge or death in hospital	
	Median (days)	Adjusted ratio (95% c.i.)*	Median (days)	Adjusted ratio (95% c.i.)*
London	9.0	1	33	1
East Midlands	6.0	0.77 (0.69,0.86)	22	0.76 (0.72,0.79)
East of England	7.0	0.78 (0.71,0.87)	25	0.79 (0.76,0.83)
North East	7.0	0.83 (0.74,0.93)	24	0.76 (0.72,0.80)
North West	7.0	0.83 (0.76,0.92)	23	0.76 (0.73,0.79)
South East	7.0	0.86 (0.78,0.95)	27	0.92 (0.89,0.96)
South West	6.0	0.77 (0.69,0.85)	30	0.95 (0.91,0.99)
West Midlands	7.0	0.79 (0.71,0.87)	22	0.79 (0.76,0.83)
Yorkshire and The Humber	6.0	0.76 (0.68,0.84)	21	0.70 (0.67,0.73)

Region	Previous revascularization		Previous minor amputation	
	Per cent	Adjusted Odds ratio (95% c.i.)*	Per cent	Adjusted Odds ratio (95% c.i.)*
London	40.4	1	16.9	1
East Midlands	39.2	0.96 (0.88,1.04)	15.2	0.93 (0.83,1.05)
East of England	36.8	0.84 (0.77,0.91)	16.8	1.00 (0.89,1.13)
North East	39.0	0.91 (0.83,1.00)	16.2	1.04 (0.91,1.18)
North West	40.8	1.00 (0.92,1.08)	16.0	1.05 (0.94,1.17)
South East	36.4	0.84 (0.77,0.91)	17.2	1.07 (0.96,1.19)
South West	39.9	0.95 (0.87,1.03)	19.7	1.14 (1.02,1.27)
West Midlands	38.0	0.89 (0.82,0.97)	19.2	1.14 (1.01,1.27)
Yorkshire and The Humber	41.1	1.03 (0.95,1.12)	15.5	0.93 (0.83,1.04)

*Rate ratio adjusted for age, sex, year and deprivation. Odds ratio for AKA also adjusted for co-morbidities. All other ratios also adjusted for co-morbidities and level of amputation. AKA, above knee amputation.

#### Regional variation in amputation rates

Amputation rates were highest in the North and much lower in London than in the other regions. Adjustment for age, sex, year and deprivation reduced rate ratios but did not substantially alter the overall geographical pattern observed (*Table 3* and *Fig. 5a*).

The percentage of major amputations that were AKA was highest in the North (55%) and lowest in the South West (42.7%). Adjustment for age, sex, year, deprivation, diabetes and co-morbidities made little difference to the overall geographical pattern observed (*Table 3* and *Fig. 5b*).

**Fig. 6:**
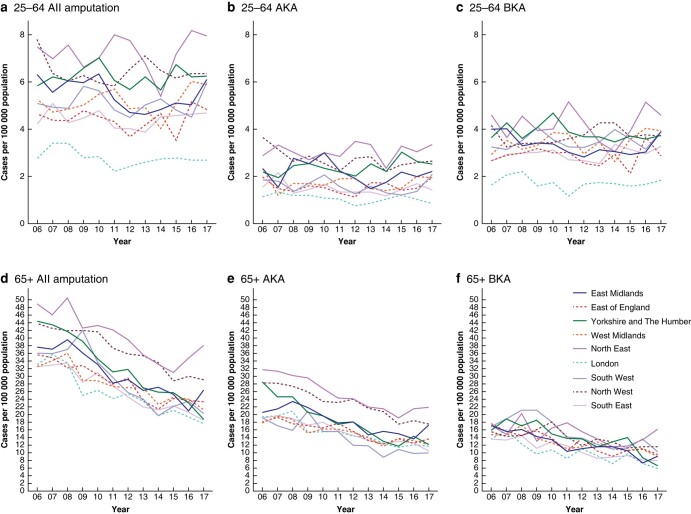
Regional time trends in population-based major amputation rates in people aged 25–64 and 65+ years; England (April 2006 to March 2018) AKA, above knee amputation; BKA, below knee amputation.

*Figure 6* shows time trends in regional rates for AKA and BKA from 2006/07 to 2017/18 in two broad age groups (25–64 and 65+ years). In the population aged 25–64 years, amputation rates were lowest in London and this was most noticeable for BKA rates. There were no clear trends over time in regional amputation rates in this age group. In the population aged 65+ years, amputation rates were highest in the North West and North East regions, and this was most noticeable for AKA rates. Rates in all regions generally decreased over time in this age group.

#### Regional variation in short-term mortality

There was generally little variation in in-hospital mortality, which ranged from 14.0% in the East of England to 16.6% in London. Ninety-day mortality ranged from 16.8% in the South West to 21.1% in the North West. Within these relatively narrow ranges, there was a suggestion that the Odds ratios for short-term mortality, adjusted for age, sex, year, deprivation, diabetes and co-morbidities, were generally higher in the North and the Midlands but also high in London (*Table 3* and *Fig. 5c, d*).

#### Regional variation in lengths of stay in hospital

Duration from admission to amputation, and from amputation to discharge or death in hospital, were both longest in London. There was also a tendency for longer duration from amputation to discharge or death in hospital in the South of England (*Table 3* and *Fig. 5e, f*).

#### Regional variation in previous procedures

The percentage of patients with previous revascularization ranged from 36.4% in the South East to 41.1% in Yorkshire and the Humber. Adjusted Odds ratios for previous revascularization were lowest in the East, South East and West Midlands and relatively high in the North and London (*Table 3* and *Fig. 5g*).

The percentage of patients with previous minor amputation ranged from 15.2% in the East Midlands to 19.7% in the South West. Adjusted Odds ratios for previous minor amputation were lowest in the East Midlands and Yorkshire and the Humber (*Table 3* and *Fig. 5h*).

## Discussion

The overall annual population-based PAD-related major lower limb amputation rate was 11 per 100 000 in the population aged 25+ years, with an overall AKA:BKA ratio of 1.03. Amputation rates were higher in men than women.

The major lower limb amputation rate was substantially higher in the population with diabetes than in the population without diabetes, with a rate ratio, adjusted for age, sex and year, of 4.82 (95% c.i. 4.73 to 4.91). The overall AKA:BKA ratio was 0.63 in patients with diabetes and 1.62 in patients without diabetes.

With regard to mortality following amputation, 25% of all patients with AKA had died within 90 days of amputation. Patients with diabetes had a median survival of only 1.68 years following AKA.

Over the 12-year time period examined, both AKA and BKA rates decreased in the population with diabetes. In the population without diabetes, there were small decreases over time. Short-term mortality, duration from admission to amputation and length of stay from amputation to discharge or death in hospital decreased over time. The percentages with previous revascularization for PAD generally increased, but the percentages with previous minor amputation tended to remain relatively unchanged over the time period examined.

Amputation rates were highest in the North of England, as was the percentage of amputations carried out as AKA, and adjustment for age, sex, year and deprivation did not substantially alter these overall geographical patterns. Adjusted short-term mortality (in-hospital and 90-day) following amputation was generally higher in the North and the Midlands but also high in London. There were also regional variations in adjusted duration from admission to amputation, length of stay from amputation to discharge or death in hospital, previous revascularization and previous minor amputation. In the 65+ age group, amputation rates generally decreased over time in all regions in England. However, in the 25–64 age group there was no clear evidence of a decrease.

The present study found that population-based AKA and BKA rates decreased over a 12-year period mainly in the population with diabetes, with minor decreases in the population without diabetes. Ahmad *et al.* similarly found a much larger decrease in the population with diabetes over a 10-year period but did not present rates separately for AKA and BKA^6^. Moxey *et al.* found no decrease in population-based AKA and BKA rates but only examined trends over a 5-year period and did not present rates separately for the populations with and without diabetes^8^. Vamos *et al.* found minor decreases in rates over time in the populations with and without diabetes but also only examined trends over a 5-year period and did not present rates separately for AKA and BKA^4^. McCaslin *et al.* examined rates over a 16-year period but calculated rates for people with and without diabetes without splitting the denominator population by diabetes status^12^. Their results are therefore difficult to interpret.

The amputation rate ratio for the population with diabetes relative to the population without diabetes adjusted for age, sex and year in the present study was 4.82. Ahmad *et al.* found an age-adjusted rate ratio of approximately 7^6^. Vamos *et al.* found an unadjusted rate ratio of approximately 15^4^. The equivalent unadjusted rate ratio from the present study was 8.86. The lower rate ratio in the present study compared with these two previous studies might be because this study covered a more recent time period and there has been a much greater decrease in major amputation rates in the population with diabetes over time. Holman *et al.* reported a rate ratio of approximately 23, but this was for major and minor amputation combined^5^ and minor amputation is a much more common procedure in people with diabetes.

The overall AKA:BKA ratio in the present study was 1.03. The National Vascular Registry reported AKA:BKA ratios of 1 and 0.98 in 2019 and 2020, respectively^15^. Moxey *et al*. reported a ratio of 1.08 while the ratio from the study by McCaslin *et al.* was approximately 1^8,12^. These are consistent with the present study. In addition, the present study also examined ratios in patients with and without diabetes, highlighting the stark contrast between these two groups with ratios of 0.63 and 1.62, respectively.

The National Vascular Registry reported in-hospital mortality of around 6% for BKA and 10% for AKA in 2019–20^15^. However, the Registry does not routinely link data to registered death records so does not provide 90-day mortality. Moxey *et al.* found that in-hospital mortality following major lower limb amputation (AKA and BKA combined) decreased from 18.6% in 2003/04 to 15.2% in 2007/08^8^. The in-hospital mortality observed over time in the present study is broadly in keeping with these results.

With regard to regional variation, the present study found clear evidence of higher major amputation rates in the North, consistent with previous studies^7,8^. This might be due to a higher population prevalence of disease, worse quality of care or a combination of these factors. The present study found this high rate remained after even adjustment for socioeconomic deprivation, which is to some extent a proxy for disease prevalence.

A higher AKA:BKA ratio and higher short-term mortality following amputation may be indicators of worse quality of care. The present study found that these two indicators were higher in the North, which has also been observed previously^8^. The higher AKA:BKA ratio and higher short-term mortality in the North found in the present study remained after adjustment for potential confounding variables, including socioeconomic deprivation and co-morbidities. This suggests that there may be issues with quality of care. The lower proportion of patients in the North who had a previous minor amputation found in the present study might also be an indicator of worse quality of care as minor amputation ought to be attempted first if appropriate.

Longer duration between admission and amputation has been found to be associated with worse outcomes^24^. Similarly, longer lengths of stay in hospital following amputation might lead to worse outcomes. However, the present study found no evidence that these indicators of quality of care were worse in the North. Revascularization should be attempted first, if possible, to avoid amputation. A low percentage of previous revascularization in patients with amputation is an indicator of worse quality of care. However, this was not found to be the case in the North. Previous revascularization was in fact relatively high in the North in the present study, a finding which has also been observed previously^7^. Overall, therefore, some indicators of quality of care suggest worse quality of care in the North, but others do not.

A key strength of using HES data is that data capture is likely to be complete, unlike the National Vascular Registry database where data capture is estimated to be 80–90% for major amputations^15^. In addition, HES data are linked routinely to mortality records, allowing more complete estimation of mortality than relying only on in-hospital deaths, which can be influenced by discharge policies over time and geographically. The complete data capture also allows more reliable calculation of population-based rates.

There are, however, a number of potential limitations which need to be considered. There may have been errors in the accuracy of diagnostic and procedure coding. There may have been changes in coding practices over time, which could also have varied geographically. As HES is essentially an administrative data set, it contains very limited clinical information. In addition, coding regarding which leg a procedure was carried out on was not reliable enough to be used.

There may have been errors in the mid-year estimates of population denominators. Estimates of diabetes prevalence could also have been imprecise. Therefore, the results need to be interpreted with a degree of caution.

The Vascular Society for Great Britain and Ireland has produced a best practice clinical care pathway for major amputation surgery^10^. The All-Party Parliamentary Group on Vascular and Venous Disease in the UK highlighted the issue of avoidable lower limb amputations and associated early death related to PAD^11^. It proposed short-, medium- and long-term actions to reduce amputation rates. The results of this study underline the importance of ensuring that these actions are pursued, and best practice universally implemented. There may also be a place for better risk assessment to inform shared decision-making, including consideration of the appropriateness of surgical intervention in those with very high predicted mortality^25^.

The contrast in AKA:BKA ratios in patients with and without diabetes indicates that separate ratios should be routinely calculated, with additional action targeted at people without diabetes to reduce the ratio to below 1. The strikingly poor survival highlighted by the 90-day and long-term mortality results indicate that at least 90-day mortality should be routinely monitored, for example in the annual National Vascular Registry reports^10^. This will require routine record linkage of mortality records to the Vascular Registry data set.

The regional variations observed indicate that further research is needed to understand why major amputation rates, particularly AKA rates, are higher in the North in order to support action to reduce these high rates. In addition, further research is needed to understand why there is a greater likelihood of AKA in women, especially older women with diabetes.

## Data Availability

The HES data used in this project may be obtained from NHS England.
